# Perspective of People With Type 2 Diabetes Toward Self-management: Qualitative Study Based on Web Crawler Data

**DOI:** 10.2196/39325

**Published:** 2023-02-02

**Authors:** Lei Hu, Xiaoyuan Jin, Yundong Li, Hongmei Wang, Dan Yang, Ziqing Zhang, Xiaoyu He, Jing Liao, Weiju Chen, Ni Gong

**Affiliations:** 1 School of Nursing, Jinan University Guangzhou China; 2 Department of Social Medicine of School of Public Health, Zhejiang University Hangzhou China; 3 School of Ethnology and Sociology, Yunnan University Kunming China; 4 Department of Medical Statistics & Epidemiology, Sun Yat-sen Global Health Institute School of Public Health Sun Yat-sen University Guangzhou China

**Keywords:** diabetes, health management, self-management, health education, patient needs

## Abstract

**Background:**

The diabetes disease burden in China is heavy, and medical standards such as diabetes guidelines are the core reference guidelines for diabetes management for health care providers and patients. However, patients’ guideline compliance is too low, which correlates with the gap between guidelines and patients’ self-management needs. Incorporating patient needs into the guideline development would reduce this gap.

**Objective:**

We sought to capture the needs of patients with diabetes for self-management in everyday situations and to clarify the contradictions and misalignments between medical standards, such as guidelines, and patient needs.

**Methods:**

This study collected crawler-based data from 4 online health communities. We selected 1605 text records collected from Chinese patients with diabetes between March 2020 and July 2020 for analysis. The text analysis applied grounded theory to separate issues that concerned patients into 3 themes, 7 subthemes, and 25 entries.

**Results:**

Altogether, 69.03% (1108/1605) of texts were related to issues concerning disease treatment (theme B) and mainly inquired about medication use (B2 and B3; 686/1108, 61.91%), including medication choice, change in medication administration, side effects, and postmedication effects. In addition, 222 (N=1605, 13.8%) texts (theme A) concerned the explanation of disease etiology and knowledge of diabetes, and 275 (N=1605, 17.1%) texts (theme C) discussed lifestyle changes and various restrictions on life brought about by the disease.

**Conclusions:**

Our findings suggest an urgent need to improve diabetes health education and guideline development strategies and to develop health management strategies from a patient perspective to bridge the misalignment between patient needs and current medical standards.

## Introduction

### Background

As a serious and complex metabolic disease, diabetes has become a challenging global health problem. In 2021, there were approximately 537 million people with diabetes worldwide, with China having the largest number of people with diabetes at 140.9 million [1]. As a major contributor to the growing burden of chronic disease, long-term treatments not only cause a disease burden of physical pain and psychosocial problems (eg, depression and anxiety) but also raise severe economic concerns for the individuals and their family, such as being unemployed, losing one’s self-care abilities, experiencing fewer social and family interactions, and making changes in lifestyle [2,3]. Thus, developing guidelines for diabetes management approaches for all patients is essential for controlling diabetes, including correcting blood glucose levels, blood pressure, and lipid abnormalities. Existing guidelines that cover diabetes care, clinical treatment, health care practices, and knowledge education have been established and developed [4,5] and have already been implemented to achieve quality diabetes care. There is strong evidence that well-established diabetes outreach programs improve patients’ treatment adherence, self-care abilities, quality of life, and happiness [6]. Guidelines are also essential for preventing and delaying diabetic complications and decreasing the significant morbidity and mortality rates associated with diabetes.

Currently, diabetes guidelines provide health care providers with suggestions that are to be followed in educating patients and act as a central reference for patient self-management. Guidelines include standards of care, and people with diabetes should follow relevant guidelines to achieve effective self-management. For example, the American Diabetes Association standards of medical care for people with diabetes cover multiple aspects of medication control [7], blood glucose goals [8], and the promotion of behavioral changes to improve health outcomes (including diabetes self-management education and support, medical nutrition therapy, physical activity, smoking cessation counseling when required, and psychosocial care) [9]. It follows that medical standards such as those found in the guidelines require patients to make radical lifestyle changes. These individuals are challenged daily with a complex set of behaviors that require adhering to a diet plan, performing proper exercise, monitoring blood sugar, and integrating all these behavioral tasks into their daily lives [10]. In this context, the implementation of medical standards, such as guidelines, by patients is highly unsatisfactory. Compliance with current diabetes guidelines is low, with rates ranging from 7.8% to 34.1% [11,12]. It has been suggested that this may be because the guidance provided by medical standards lacks a life perspective and is somewhat detached from the daily needs of patients. This reduces both the comprehensiveness and relevance of the guidelines and universality of their application [13]. The content of current guidelines is too specialized for patients to understand and apply, as they generally contain large numbers of calculation formulas or intakes of specific nutrients (eg, “calculate nutrient ratios in foods and overall energy intake, daily salt, dietary fiber intake, etc”), which are somewhat detached from patients’ daily lives. In addition, to control the content comprehensiveness with high quality, most guidelines are prepared using expert consensus and questionnaires filled in by professionals for data collection [14]. Patient information obtained in these ways has often been limited by the medical profession, creating an overly rational and inherently standardized framework of guidelines designed to solve all patient’s problems with only a single set of protocols or formulas. Consequently, the content involved in these guidelines ignores essential information for patients, which results in a deviation from the actual needs of patients.

However, influenced by factors such as their social and cultural environments, patients return to their daily lives for disease self-management, and the daily real-life needs are individualized. In addition, patients present with various psychological states and needs at different stages of their disease. Moreover, the needs of patients with limited knowledge about their disease and its management are defined by previous studies, including clinical treatment, treatment of comorbidities, and psychosocial needs, from their limited knowledge [15]. It has been suggested that incorporating patient needs into the development of disease guidelines ensures greater validity of the guidelines and helps patients manage the progression of their condition [16,17].

### Objective

Therefore, to identify the real needs of patients with diabetes for self-management in their daily lives and to clarify the contradictions and misalignments between medical standards, such as guidelines, and patients’ needs, this study used a web data crawler method to obtain the actual content of patients’ inquiries on a web platform as a way to uncover and explore what exactly are the most important concerns of patients with diabetic in their real lives. In this way, the content of diabetes guidelines can be improved from the patient’s point of view; the educational role of guidelines can be strengthened; and truly effective 2-way health education can be implemented.

## Methods

### Ethics Approval

This study was approved by the Ethics Committee of the First Hospital of Jinan University (number KY-2022-110), and its implementation process strictly complied with the Declaration of Helsinki.

### Sampling

**Figure 1:**
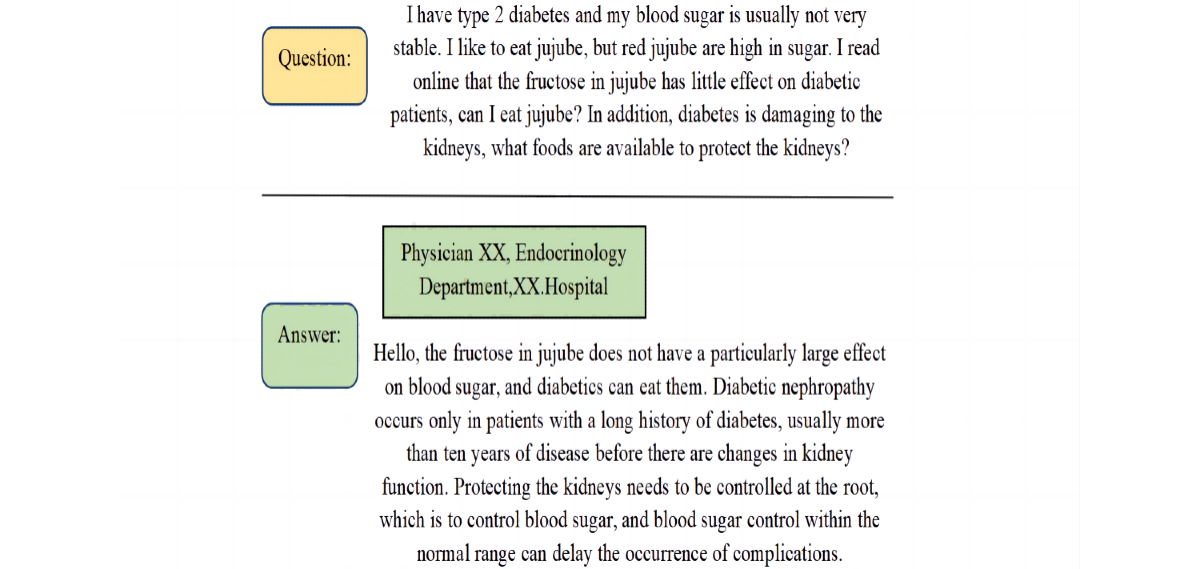
Schematic diagram of the doctor-patient conversation segment of the web crawler data.

This web crawler survey obtained 4079 crawler data points recorded between March 2020 and July 2020 from 4 web inquiry platforms. The data collected by the web crawler were in the form of question-and-answer exchanges between a patient with diabetes and physician about the term *diabetes* (Figure 1).

### Data Collection

On par with the development of digital health globally [18], the State Council of the People’s Republic of China has issued the authorized document “Opinions on Promoting the Development of ‘Internet + Medical Health’” and, since 2018, has been progressing considerably in developing information technology and health care services [19]. Notably, online health communities (OHCs) are among the essential electronic health services in contemporary society. In recent years, the use of OHCs has become prevalent among patients with chronic diseases and the application of social network analysis to research user behavior in OHCs has developed gradually [20]. In OHCs, people with diabetes browse the website for information about physicians before selecting one for medical consultation to obtain health information about diabetes and its treatment. Therefore, this study selected 4 OHCs randomly based on multiple authorized rankings of popular OHCs in China (Table 1) and archived data on patients’ web-based activities and interactions with physicians. Using a web crawler, we crawled the text of conversations between patients with diabetes and physicians from the 4 OHCs using the keyword “diabetes.”

**Table 1:** Summarized information about the 4 selected OHCs^a^.

OHCs	Ranking (monthly active member)	Web-based business services (specific services provided)								Health care providers
		Clinical treatment	Medical care (physical examination and aesthetic medicine)	Health management (health care education)	Shopping center (Health supplements)	Insurance services	Health IT	Pharmacy shopping		
A	Top 1 (11.87 million)	✓^b^	✓	✓	✓	N/A^c^	N/A	N/A	Physicians can work in any hospital, but all pharmacists have to be the official staff of company A.	
W	Top 4 (4.30 million)	X^d^	N/A	N/A	N/A	X	X	X	Physicians have to be the official staff working in company W; all specialists work in the first-class 3A hospitals.	
C	Top 3 (5.43 million)	—^e^	—	—	—	N/A	N/A	N/A	All specialists work in the first-class 3A hospitals.	
D	Top 7 (2.20 million)	—	—	—	—	N/A	N/A	N/A	All specialists work in the first-class 3A hospitals.	

^a^OHC: online health community.

^b^Teleconsultation and personal physicians.

^c^N/A: not applicable.

^d^Teleconsultation and appointment schedule system.

^e^Teleconsultation.

### Data Screening

Web-based questions from either patients with type 2 diabetes or their families were eligible for inclusion in this study. We excluded the following: (1) web-based questions from patients with type 1 diabetes, gestational diabetes, or another type of diabetes; (2) exchanges with incomplete information or unclear statements; and (3) questions resulting in ineffective communication with the physician.

### Text Analysis

**Figure 2:**
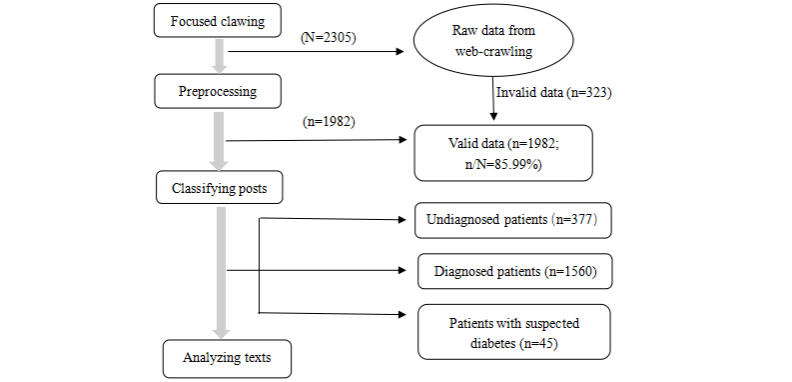
Flowchart of the data-collection process that applied web-crawler technology to data from 4 online health communities (OHCs).

This study involved an analysis of the web crawler’s web-based consultation texts of patients with diabetes, that is, patients’ web discourse. First, we qualitatively coded and analyzed the text obtained by the web crawler using the rooting theory approach and then subjected the qualitatively coded text to frequency statistics. Because the information in patients’ consultation texts in the OHCs was geographically extensive, large in content (4079 texts), and fragmented, the rooting theory proposed by Corbin and Straus [21] was used to guide the text analysis. The coding stages were divided into following three main stages: (1) open coding, (2) axial coding, and (3) selective coding. Initially, we performed line-by-line open coding from small blocks of text to identify and conceptualize personal stories and experiences of people with diabetes as keywords, obtaining 2305 codes. Among these 2305 codes, although the primary concern of all questions was related to diabetes, the types of people who asked the questions were diverse. With the support of multiple clinicians, this study divided all the questions into the following three groups according to patient characteristics: (1) undiagnosed, (2) diagnosed with diabetes, and (3) suspected diabetes (patients who had been diagnosed but did not believe that they had the disease and repeatedly confirmed it). As mentioned previously, this study sought to explore the self-management needs of patients with established type 2 diabetes. Therefore, the sample for this study included only codes related to patients with confirmed diabetes (n=1560) and physician-confirmed diabetes (n=45), resulting in 1605 codes (Figure 2).

Axial coding in the second step was used to establish the connection between the 1605 conceptual genera. The open coding results were grouped to identify the health care needs associated with diabetes and the way they change over the course of the disease. Finally, after identifying the patients’ perceived needs as a tentative core, selective coding was performed. We integrated the relationships among all extracted classes by deriving an integrated and highly abstract core class to comprehensively explain the needs of patients with diabetes during their illness and self-management.

## Results

### Overview

We found that the self-management needs of patients with diabetes could be categorized into 3 first-level codes, 7 second-level codes, and 25 third-level codes (note that because of space limitations, only the category distribution of primary and secondary codes is given; Table 2). The 25 tertiary codes are presented in Multimedia Appendix 1. Of the 1605 primary codes, 1108 (69%) were related to disease treatment problems (B), mainly medication (B2 and B3; 686/1108, 61.91%), including drug selection, change in medication administration, drug side effects, and postmedication effects. Thus, medication and treatment are the most important needs of patients. In addition, 13.8% (222/1605; A) of codes were related to the explanation of the etiology of the disease and knowledge about diabetes, whereas 17.8% (275/1605; C) of codes concerned lifestyle changes and various restrictions on life brought about by the disease. This study considered the journey from the time point of patient’s diagnosis of diabetes to the stage of treatment and self-management so that the needs that arise during the course of the disease and treatment can be explored.

**Figure 3:**
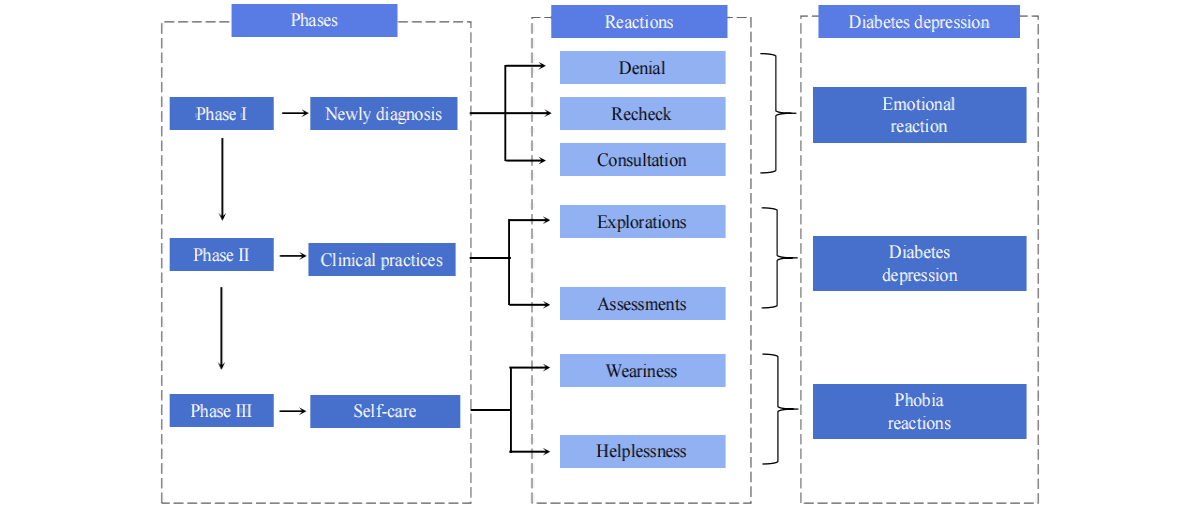
Reactions and emotional distress of Chinese patients with diabetes in different phases.

From the themes and subthemes, it is clear that different psychological and need changes exist among patients with diabetes at different stages of their disease, and this dynamic trend of changes over time is reflected in 3 stages after the disease is identified, that is, the newly diagnosed stage, the clinical treatment stage, and the self-management stage (Figure 3). Initially, patients will experience a sense of fatalism, from denying a new diagnosis to fumbling and struggling to self-manage the disease according to medical standards, and finally they are in a dilemma and fear losing control of their lives.

**Table 2:** Summarized information of themes under the grounded theory analysis (N=1605).

Themes and subthemes		Codes, n (%)
**A. Diagnosis—emotional reactions (n=** **222, 13.8%)**		
	A1. Why me?	71 (4.4)
	A2. What is diabetes mellitus?	151 (9.4)
**B. Treatment—diabetes-influenced depression (n=** **1108, 69%)**		
	B1. Is the current treatment necessary?	422 (26.3)
	B2. Why is disease management so complex?	228 (14.2)
	B3. Why does the medicine not work?	458 (28.5)
**C. Self-care—phobia reactions (n=275, 17.2%)**		
	C1. Why is daily self-management complicated?	195 (12.1)
	C2. Can I get rid of diabetes?	80 (5.0)

### Phase I: New Diagnosis—Emotional Reactions

#### Overview

Patients with diabetes were initially puzzled that they had the disease and believed that they could not have the disease. In total, 4.4% (71/1605; A1) texts in this study were related to the question “Why do I have diabetes?” When patients became aware of the fact that they had the disease, they began to ask questions about diabetes, and 9.4% (151/1605; A2) texts were related to “what is diabetes?”

#### A1. Why Me?

Once diagnosed with diabetes, the patient may express their anguish about having such an unexpected clinical condition. In addition, they may become frustrated as to why they have diabetes:

I never smoke and drink in my daily life, but why was I diagnosed with diabetes?

I went to the hospital for a physical examination last month. The physician told me that I had diabetes, but I never eat sweets [and] even only have a few drinks. How could I get this disease?

#### A2. What Is Diabetes Mellitus?

Patients with diabetes may refuse to believe in the diagnostic methods or report, avoid the thought of long-term complications, and choose to confirm the accuracy of the clinical examination results repeatedly by asking different physicians (A2.1):

The results of my two clinical examinations are different. I am wondering how likely [it is that] the results are [wrong]? To double-check, before doing the physical examination, the only pre-request is a fasting state, right? Also, having stayed up last night would not affect the examination results, right?

The results from physical examinations taken in the hospital diagnosed me with diabetes. Is the glucose tolerance test accurate to diagnose diabetes?

After being diagnosed by multiple physicians in the OHCs, the patients gradually realized the facts of their disease. They began to ask more about diabetes-related information, especially about gestational diabetes mellitus and hereditary factors (A2.2):

I have GDM. If I have a child in the future, will him/her [my next generation] be influenced because of the hereditary factors?

The father has diabetes and needs supplemental insulin therapy. Will it be inherited? How likely is the child to have diabetes?

After the patients have a preliminary understanding of diabetes, they begin inquiring about consultations with physicians, including the associated costs and clinical plans during the treatment process (A2.3 and A2.4):

I am a newly diagnosed patient with diabetes, and I want to ask about the treatment methods and relative costs.

### Phase II: Treatment—Diabetes-Influenced Depression

#### Overview

Over time, the patients finally accepted their situation and prepared themselves for living with diabetes. After accepting the validity of their diagnosis, patients often sought information regarding diabetes treatment. However, as it is a lifelong chronic disease, treatment involves long-term comprehensive health management. When the treatment effects could not be observed immediately and directly, patients were in doubt about the existing clinical treatments and asked the physicians to revise their treatment plans. However, the desired effects under treatment were difficult to achieve. Thus, patients experienced dissatisfaction and doubt throughout the treatment process. In this study, 26.3% (422/1605; B1) included texts doubting the current perceived treatment, asking “should this be treated?”; 14.2% (228/1605; B2) texts expressed worries such as “why the daily diabetes management is so complex”; and 28.5% (458/1605; B3) involved patients’ self-assessment of the treatment effects and asked, “why taking medicine does not work?”

#### B1. Is the Current Treatment Necessary?

After initially accepting the existence of the disease and entering the treatment phase, some patients had doubts about their existing treatment plan (B1), including whether the standardized treatment provided by doctors was reliable (B1.1), whether lifestyle changes were functional (B1.2), and the treatment of complications and comorbidities (B1.3):

After taking medicine, my mother felt her hands and feet still were numb and weak. I want to ask about [whether] the current medication use is reasonable or not? Can I ask to modify the current treatment plan?

When the blood glucose management remained suboptimal for a long-term period, some patients even tried to seek home remedies, unproven alternative medicines, and unvalidated treatments (B1.5). Specifically, patients with chronic illness or long-term poor blood sugar control were more likely to be drawn to the “magic effects” of alternative medicine and treatments:

Can patients with type 2 diabetes drink homemade enzymes? Does it help [to treat] diabetes?

I heard [that] electromagnetic therapy can cure diabetes, but I dare not try it rashly. [So], doctor, what is the principle of electromagnetic therapy? Can it treat my diabetes?

#### B2. Why Is Disease Management So Complex?

For patients with diabetes, daily disease management included medication and blood sugar monitoring. Our text analysis revealed that the patients were most concerned about medication issues. In particular, patients asked more about the need to take pills and the choice of and the matching types of medications at their initial stage of medicine use or when their disease symptoms were not apparent (B2.1):

[I was diagnosed with] diabetes one or two years ago. After taking medicine for a while, [my blood sugar] became stable. After stopping the medicine, it remained stable, but today, the exam result found [that my] blood sugar was increased, even to 8.1. Under this condition, should I take medicine?

Patients also expressed different needs according to the 2 significant situations that could arise after taking the medication. Patients with reasonable blood sugar control hoped to stop their medication intake after achieving a stable blood sugar level (B2.2). In contrast, patients who did not experience an efficient medicine effect tended to ask or request to adjust the dosage or change their current drugs (B2.3). Some also asked about drug side effects (B2.4):

[I was diagnosed with] diabetes half a year ago. I had kept taking medication in the last period. [My] fasting blood sugar was 7.2, and the postprandial blood sugar was normal [when I did not take medicine], so I self-decided to stop taking medicine. [Does] this situation express the control of my blood sugar pretty well? Can I stop taking medicine then?

I took the short-acting pills of Novoline R [Novo Nordisk, Bagsværd, Denmark] two months ago. Since I had hypoglycemia frequently at night, the outpatient physician changed (my pills) to Humalog [Eli Lilly and Company, Indianapolis, IN, USA], but I did not feel it was efficient in controlling [my blood sugar]. Thus, I am curious [about] which one is better?

Patients with diabetes must take medication for their entire lives, and most of them were aware of this, but many patients still repeatedly ask questions about their medications, while the equally important issue of blood glucose monitoring is rarely posed. Only a few patients mentioned the method of blood sugar monitoring (B2.5) or evaluation of clinical test results (B2.6):

I rarely test my blood glucose, but if my blood glucose is high, the smell of my urine changes immediately, and then I test my blood glucose. Yesterday, my fasting level was 7.55. Is this a normal blood glucose level?

#### B3. Why Does the Medicine Not Work?

Generally, patients believed that medication can be effective in disease treatment. However, in some cases, long-term medication use did not have the desired therapeutic effect or even worsened the patient’s condition, intensifying their doubts about the current treatment options. This was especially true for poor blood sugar control (B3.1) or the occurrence of diabetic complications (B3.2) after medication use:

I take metformin and acarbose regularly every day, but my fasting blood sugar cannot be lowered to 7.8. What should I do?

When taking medicines for a long time, patients tended to wonder whether diabetes was the cause of any unpleasant symptoms they felt (B3.3):

I have diabetes—is the blister on my foot poisonous gas coming out?

It’s scary when you have frequent hypoglycemia and sweating. Sometimes, [I feel] delirious, sweating desperately—what should I do?

### Phase III: Self-care—Phobia Reactions

#### Overview

Maintaining a healthy lifestyle routine is essential for patients with diabetes. However, some patients felt distressed by the complex management of diet and exercise and believed that the various restrictions brought about by diabetes management seriously affect their everyday lives. For example, 12.1% (195/1605; C1) of texts asked questions such as, “why is daily self-management so complicated?” A small number of patients also realized that because diabetes was a chronic disease, a poor lifestyle could exacerbate their condition, and they expressed a desire to improve their health by changing their daily routines. However, when patients did not see efficacy after undergoing long-term treatment or they realized that current clinical practices could not treat diabetes, they reacted with fear; indeed, 5% (80/1605; C2) of the codes were related to questions that were some variation of “can I get rid of diabetes?”

#### C1. Why Is Daily Self-management Complicated?

Patients self-managed their diabetes by controlling their diet (C1.1) and performing reasonable amounts of exercise (C1.2). However, during complex diet and exercise management period, patients realized that their disease had brought various inseparable restrictions to their everyday lives (C1.3):

I’m a foodie, and I have a hard time when I don’t get a meal that I like. I know I have to control my diet, but I can’t stop thinking about good food. Especially when I eat at a restaurant at noon on a weekday, I order a few things I love and then eat them all, and then I find that my blood sugar is out of control after I eat.

It’s too hard to control [your diet] in life. People ask you to drink some wine. How can you do that when you say you won’t drink anymore? This is also rude to others. I can only say that I have diabetes, try to drink as little as possible, and only drink two glasses.

I work every day, and often work overtime. [I am] already very tired, and the doctor said that I have to exercise every day, and I really cannot eat. What should I do?

When I took insulin, people who didn’t understand thought I was on drugs. Then, I didn’t want people to see me taking insulin, so I secretly took it inside my room by myself.

#### C2. Can I Get Rid of Diabetes?

Because of the severe limitations on daily life and incurable nature of diabetes, patients often suspected that diabetes would shorten their life span (C2.1) and were concerned that the disease could not be cured (C2.2):

Is diabetes scary? Is it true that diabetes cannot be treated? I got diabetes when I was 43 [years old]. How will I live in the future? I just want to know, [are] there any clinical practices to treat diabetes completely right now?

How much does diabetes affect life expectancy?... Since I am only 34 years old now. From the Internet, I realized that diabetes always develops into kidney disease and kidney failure, whether it is earlier or later. Is it true? Some people also say that most patients with diabetes cannot live beyond 25 years.

## Discussion

### Principal Findings

The results of this study suggest that the needs expressed by patients from the time point of diabetes diagnosis to the entire course of disease treatment are dynamic. We captured the patients’ changing psychology and needs during this dynamic process of evolution from denial to acceptance to questioning and finally coming to a standstill. From the patients’ perspective, we find that their feelings and needs at different stages are misaligned with medical standards, and this misalignment between daily life and medical standards is the inner logic of the gap in patients’ self-management behavior. In the discussion that follows, we summarize the internal logic of dynamic self-management needs in 3 areas.

### Conflict: Overidealization and Disruptions to Daily Life

In the disease treatment process, people with diabetes are reluctant to believe that they have the disease in the beginning [22,23]. After learning about diabetes and its treatment, patients find that they need to make radical lifestyle changes, and they are challenged daily with a complex set of behaviors that require following a diet plan, performing proper exercise, monitoring blood sugar, and integrating all of these behavioral tasks into their daily lives [24]. Long-term life behavioral changes can be a considerable challenge for many patients. Over time, they hope to return to their familiar lifestyle as soon as possible through a treatment program that does not disrupt their old lifestyle. As found in this study, they prefer to place their expectations on medications [25]; other nondirective treatments such as exercise, blood glucose monitoring, and diet control are rarely on their list of concerns [26]. In other words, the patients felt that taking drugs is easier than changing their lifestyle behavior [27]; patients are more likely to expect a “magic pill” that will cure them once and for all or at least maintain their relative health status.

Lifestyle changes form an important foundation for current diabetes management. However, lifestyle changes and daily management can be difficult for patients. The most basic self-management measures of diet control, exercise, and blood glucose monitoring can cause patients to feel disrupted [10,28]. This study found that patients generally showed poor compliance with blood glucose monitoring. Some patients do not monitor their blood glucose levels to avoid the consequences of elevated blood glucose owing to relaxed dietary control, choosing to adopt this “cover-up” approach to avoid predictable adverse outcomes. In addition, they use physical sensations as a yardstick for blood glucose levels and only monitor blood glucose when they are unwell (eg, when they experience changes in urine smell, dizziness, etc) [29]. The results of this study showed that many patients did not have a well-established health management system. Blood glucose monitoring are not well embedded into their diet and lifestyle behaviors and are decoupled from their daily lives, making the small but important task of blood glucose monitoring a big burden.

### Opposition: Everyday Logic and Medical Rationality

The medical standard, as a medical rationale, attempts to medicalize a patient’s daily life by requiring a complete change in their lifestyle. Medical rationale has a systematic explanation and response plan for the development of diabetes, such as self-management for patients. The following seven self-management behaviors recommended by the American Diabetes Association provide an evidence-based framework: (1) healthy eating, (2) active exercise, (3) blood glucose self-monitoring, (4) medication, (5) problem-solving, (6) reduce the risk of complications, and (7) healthy coping [30]. This scientific approach is the only way to control the disease. Medical rationality treats the patient as a “rational, perfect patient,” whereas the patient themselves plays many social roles in addition to a patient role, and their disease is just one aspect of their daily lives. In other words, medical rationality lacks a life perspective and ignores the patients’ social attributes. For patients who are accustomed to their original lifestyle, maintaining their original lifestyle is a symbol of maintaining a relatively healthy state [31]. Patients will grasp autonomy according to their individual feelings, and they espouse a logic of life that restores and maintains a long-established way of life. Patients are forced to integrate the demands of medical rationality into their daily lives, which have an inherent form. The conflict between patients’ daily lives and medical rationality results in low levels of self-management, difficulty finding a balance between life and illness, and even less success in integrating illness and its management into daily life and situational interactions.

In this study, the conflict between medical rationality and life logic is demonstrated by the fact that patients have a correct understanding of the importance of life behavioral changes but lack action. In Chinese food culture, the Chinese table is an important vehicle for social interactions. Doing business negotiations, visiting friends and relatives, and maintaining social relationships are all important dietary tests for patients with diabetes. This study found that patients believe that enjoying their preferred food is an indispensable and wonderful experience in life and that a life of controlled eating would lose its wonderful meaning. This is similar to the findings in the study done by Tan et al [32], who reported that Chinese patients with diabetes believe that it is pointless to control their diet, especially on special occasions such as holiday celebrations. In addition, China is a multiethnic country with numerous cuisines. Under the influence of this social environment and food culture, the dietary control of patients with diabetes is weakened. Performing regular physical exercise can be a huge challenge for patients. Among young patients, the pressure of working overtime and the increasing pressure of life leave them with little time to exercise. Previous studies have also demonstrated that work is a major barrier to physical exercise in men [33]. Relatively, older patients have more leisure time to exercise; however, their physical condition does not enable them to exercise regularly, and some patients report having multiple diseases, heart surgery, or difficulty with their legs, which make it difficult to exercise consistently. It follows that the focus of patients is on trying to live a normal life, whereas that of medical rationality is on wanting the patient to live a normal life without jeopardizing their diabetes management. In turn, this can result in a contradiction between the patient’s desired treatment outcome and the reality of the disease.

### Misalignment: Actual Patient Needs Versus Current Health Education and Guidelines

The opposition between the actual needs of patients and the standards of care can be seen in the dichotomy between everyday logic and medical rationality. The needs of patients throughout the treatment stage are related to real-life daily needs, and they hope to receive more *everyday* guidance from their physician. Patients with overidealized views are in pursuit of immediate short-term treatment effects, hoping to quickly return to normal life without changing their lifestyle. In addition, owing to the complexity of diabetes itself and individual differences in the affected population, the needs of patients with diabetes are highly unique [34]. The treatment and drug-related issues that patients are most concerned with require detailed analysis, including drug replacement, if required; adjustment of medication; and guidance regarding the effects and side effects of drug use. For the short- and long-term complications of diabetes, patients need to have detailed relevant knowledge to deal with various symptoms that are difficult to cope with in daily life. Many patients also find it difficult to cope with fluctuations in blood sugar and deterioration of their condition after medication and require more specific guidance and support. This study found that patients try alternative medicines or alternative treatments, such as herbal medicines and unproven treatments, and even believe that prescriptions obtained from relatives or on the internet may be more effective.

The content of current diabetes guidelines is typically an overly rational and inherently standardized framework that aims to solve all the problems of patients using only 1 set of programs or formulas, although diabetes management includes disease diagnosis, diet, exercise, drug treatment, blood sugar monitoring, and psychological interventions. There are considerable differences in the specific issues that patients care about in each dimension; however, existing guidelines only prepare a guidance plan for each dimension based on standard issues. Guidelines are standardized, but patients have individual needs, so the actual needs of patients and current diabetes health guidelines are also different [16].

Therefore, to ensure the authority of medical standards, such as guidelines, and the optimization of guidelines, it is necessary to minimize or eliminate the discrepancies between actual patient needs and current medical standards. The incorporation of patients’ needs into the process of preparing medical standards, such as guidelines, will more comprehensively reflect the patients’ problems and resolve their most important concerns to further improve disease prevention and management.

### Realistic Significance

To address poor disease management, a single effort to develop the health care system is not enough, that is, the needs of the patient audience are another important aspect of facilitating development. First, this study focused more on the needs of patients in the real world and found discrepancies between the guidelines and patient needs. This provides a basis for the development of future medical standards, including guidelines. Second, this study provides a new dissection of the gap in the physicians’ understanding of the patient’s needs in their daily lives. Finally, our study facilitated communication between physicians and patients. Studying patient needs from the patient’s perspective facilitates health care providers to pay more attention to health care personalization and customization [35] and patients’ real-life problems and needs and to have a foresighted prognosis of patients’ problems.

### Limitations

This study has some limitations. First, there may be some shortcomings inherent to the qualitative analysis of text using a web crawler, which is a new attempt by our team. Second, this qualitative study collected real data from patients’ questions for analysis and discussion, so some of the original words said by patients were quoted in the discussion for interpretation, which may be slightly subjective. Third, our analysis relies only on OHC web crawler data and misses a variety of other information, such as sociodemographic characteristics and insurance coverage. Therefore, applying the main findings of this study as a preliminary basis, future studies may consider the patient population characteristics in depth and conduct a more detailed analysis by interacting with patients.

### Conclusions

This study explored the health management needs of patients with diabetes in real-life situations during their disease and treatment journey using web crawler data. The needs of patients during their illness and their internal logic may be summarized in the following 3 dimensions: conflict between overidealized views and disrupted daily life, opposition between everyday logic and medical rationality, and misalignment between patients’ actual needs and current health education. The fundamental solution to poor disease control is to resolve these conflicts, oppositions, and misalignments. Our findings suggest that there is an urgent need to improve strategies for developing standards of care, such as diabetes guidelines, and to develop health management strategies from a patient perspective to bridge the misalignment between patient needs and current standards of care.

## Appendix

Multimedia Appendix 1 Summarized information about the 4 selected online health communities.

## Abbreviations

OHC: online health community

## Data Availability

The collected data files are available upon request from the corresponding author NG.
