# Supplementary material for: Perspective of People With Type 2 Diabetes Toward Self-management: Qualitative Study Based on Web Crawler Data
Source: J Med Internet Res. 2023 Feb 2;25:e39325. doi: 10.2196/39325 (PMC9936364; doi:10.2196/39325)
Supplement: Multimedia Appendix 1 [file jmir_v25i1e39325_app1.docx]

Multimedia appendix 1

Table S1. Summarized information about the four obtained OHCs.

| **OHCs** | **Ranking (Monthly active member)** | **Online business services (Specific provided services)** | | | | **Healthcare Providers** |
| --- | --- | --- | --- | --- | --- | --- |
| A | Top 1  (11.87 million) | Clinical treatment (Teleconsult-ation; Personal physicians) | Medical care (Physical examination; Aesthetic medicine) | Health management (Health care education) | Shopping center (Health supplements) | Physicians can work in any hospital, but all pharmacists have to be the official staff of company A. |
| W | Top 4  (4.30 million) | Clinical treatment (Teleconsul-tation; Appointment schedule system) | Pharmacy shopping | Insurance services | Health Information Technology | Physicians have to be the official staff working in company W; all specialists work in the First-class 3A Hospitals. |
| C | Top 3  (5.43 million) | Clinical treatment (Teleconsult-ation) | Medical care | Health management | Shopping center | All specialists work in the First-class 3A Hospitals. |
| D | Top 7  (2.20 million) | Clinical treatment (Teleconsult-ation) | Medical care | Health management | Shopping center | All specialists work in the First-class 3A Hospitals. |
